# Gut Microorganisms, Neuroinflammation and Behavioral Changes

**DOI:** 10.1192/j.eurpsy.2024.1297

**Published:** 2024-08-27

**Authors:** B. T. Adebisi

**Affiliations:** Functional and Molecular Neurobiology, Institute of Anatomy, Cell Biology, Brain and Neurodegeneration, Osogbo, Nigeria

## Abstract

**Introduction:**

Recent clinical and preclinical evidences suggested that neuroinflammation is a key factor which interacts with the neurobiological correlates of major depressive disorder, which are the (i) dysregulation of the hypothalamic-pituitary-adrenal axis, (ii) depletion of brain serotonin and (iii) alteration of neurogenesis in the dentate gyrus of the hippocampus.

The gut bacterial has major impact on the brain development, behaviour and host immune system through the microbiota-gut-brain axis.

**Objectives:**

The objective of the research is to establish the role inflammation induced by gut dysbiosis plays in behavioural changes of patients suffering from major depressive disorders.

**Methods:**

Clinical data and preclinical experiments were used to elucidate the role gastrointestinal bacterial play in the development and functional physiology of the nervous system and because of the bidirectional communication between the enteric nervous system in the gut and the central nervous system, through the vagal plexus, blood circulation and endocrine system; it was discovered that the appropriate population of intestinal microbiota affect the immunological state of the brain.

**Results:**

The intestinal microbiota has been able to maintain the attenuation and regulation of pro-inflammatory biomarkers in the brain and such had assisted in the healthy state of the brain; however, a disruption of gastrointestinal organisms in a condition called dysbiosis could result in breakdown of protective gastrointestinal mucosa barrier resulting in leaky gut and consequently, the permeability of the gut lining and migration of some bacteria, to the brain through the vagal networks and other channels.

These pathophysiological cascades appear to be triggered or sustained and reinforced by chronic inflammatory condition involving increased circulating markers of inflammation, which are able to cross the blood brain barrier to activate the microglia.

**Conclusions:**

Studies in depression suggest that inflammatory biomarkers such as C-reactive protein can be used to enrich samples for anti-inflammatory clinical trials for depression that target inflammation-related symptoms such as anhedonia and anxiety.

Although, still at the developmental stages, imaging of neuroinflammation will help establish a target in the brain to further facilitate the testing of anti-inflammatory therapies for depression.

**Disclosure of Interest:**

None Declared

